# A lack of information engagement among colorectal cancer screening non-attenders: cross-sectional survey

**DOI:** 10.1186/s12889-016-3374-5

**Published:** 2016-07-29

**Authors:** Lindsay C. Kobayashi, Jo Waller, Christian von Wagner, Jane Wardle

**Affiliations:** Department of Epidemiology and Public Health, Health Behaviour Research Centre, University College London, 1-19 Torrington Place, London, WC1E 6BT UK

**Keywords:** Colorectal cancer screening, Faecal occult blood test, Population screening, Decision making, Public education, Survey, England

## Abstract

**Background:**

The NHS Cancer Screening Programmes in England now operate a policy of ‘informed choice’ about participation in cancer screening. Engagement with written information about screening is important to facilitate informed choice, although the degree to which the screening-eligible public engages with the available information is unknown. We examined the association between reading of the standard informational booklet (‘*Bowel Cancer Screening: The Facts*') and participation in the nationally organised NHS Bowel Cancer Screening Programme in England.

**Methods:**

Face-to-face interviews were conducted with 1307 adults who were age-eligible for nationally organised colorectal cancer (CRC; also called bowel cancer) in a population-based survey in England in 2014. Respondents were shown an image of *‘The Facts’* booklet and were asked how much of it they had read when they received their screening invitation (‘none’, ‘a little’, ‘some’, ‘most’, ‘almost all’, or ‘all’). Logistic regression was used to estimate the associations between screening uptake status (‘never’ vs. ‘ever’) and self-reported reading of *‘The Facts’* booklet (dichotomised to ‘none vs. ‘any’), adjusting for age, sex, ethnicity, educational attainment, and occupation-based social grade.

**Results:**

Overall, 69 % of the sample (908/1307) had participated in CRC screening at least once (‘ever’ screeners). One-fifth of the sample reported that they had read ‘none’ of *‘The Facts’* booklet (22 %; 287/1307), while half reported having read ‘all’ of it (52 %; 680/1307). Reading of the booklet was strongly differential according to screening uptake status: nearly two-thirds of ‘never’ screeners had read none of *‘The Facts’* booklet (63 %; 251/399), compared to less than one in twenty ‘ever’ screeners (4 %; 36/908); adjusted OR = 39.0; 95 % CI: 26.2-58.1 for reading ‘none’ in ‘never’ vs. ‘ever’ screeners.

**Conclusions:**

Although *‘The Facts’* booklet is intended to support informed choices about CRC screening, the majority of unscreened individuals report that they have read none of it. The degree to which public engagement with the decision-making process about cancer screening is socially unequal must be better understood so that comprehensive and equitable public communication strategies can be developed.

## Background

In England, home-based screening for colorectal cancer (CRC, or, bowel cancer) with the faecal occult blood test (FOBt) is publicly available through the National Health Service (NHS) Bowel Cancer Screening Programme. Screening was introduced in pilot areas from 2006 for men and women aged 60-69 years and was fully implemented nation-wide with the upper age limit extended to 74 years from 2010 [1]. Men and women are invited to screening on their 60^th^ birthday and biennially thereafter. The screening invitation consists of a letter that arrives in the post with the information booklet ‘*Bowel Cancer Screening: The Facts*’ [2]. The FOBt kit and instructions booklet arrive in the post one week later. *‘The Facts’* booklet is intended to provide necessary information about the possible benefits and harms of FOBt-based CRC screening and to guide informed decision-making among screening-eligible adults, in advance of receipt of the actual screening test kit. Although ‘ever’ screening uptake is 70 % among the original cohort of adults invited to screening by the NHS, only 58 % of the total screening-eligible population was adequately screened in 2012-13 [3, 4].

Low accessibility of the written screening information for people with poor literacy or health literacy skills may partly contribute to low uptake of FOBt screening in England. Health literacy is defined as the ability to obtain, process, and understand health-related information to make informed health decisions [5]. Low health literacy has been associated with low uptake of FOBt screening for CRC in England and elsewhere [6–11]. Low health literacy is associated with a greater burden of information processing when reading informational materials about CRC screening, and a lower awareness of and knowledge about CRC screening [8, 12–15]. Low health literacy has been associated with a low degree of informed decision-making about CRC screening, although evidence is only just accumulating in this area [16–18]. Some research indicates that informed decision making is low overall among screening-eligible adults, regardless of literacy levels [17].

These relationships between health literacy and CRC screening-related outcomes are potentially problematic for the NHS Bowel Cancer Screening Programme in England. Although the programme effectively reduces transportation and time barriers that may exist for screening tests that are clinic-based, it is heavily reliant on written information sent through the mail for public communication. This design places onus on individuals to read, comprehend, and make a decision based on the written materials, with no input from a health professional. Although the *‘Bowel Cancer Screening: The Facts’* booklet is approved by the United Kingdom (UK) Plain English Campaign, it is associated with an estimated literacy level for comprehension that is achieved by only 57 % of the English population aged 16 to 65 years [19, 20]. Fewer adults aged over 65 years are expected to be at this literacy level, as literacy and cognitive skills are lower, on average, among this age group than among younger adults [19, 21]. The standardised, written information distributed by the NHS may therefore be inappropriate to engage a substantial proportion of the target public and enable them to make informed decisions about CRC screening.

We examined the association between self-reported reading of ‘*Bowel Cancer Screening: The Facts*’ and participation in nationally organised CRC screening in England among screening-eligible men and women aged 60 to 70 years old.

## Methods

### Design

The population-based Attitudes, Behaviour, and Cancer UK Survey (ABACUS) was conducted in England between January and March 2014 by TNS Research International. The survey methods are described in detail elsewhere [22]. Briefly, random location sampling points were created from the 2001 Census small-area statistics and the Postcode Address File, stratified by social grade and Government Office Region. At each location, quotas were set for age, gender, children in the home, and working status. Survey respondents were asked to voluntarily participate in a face-to-face computer-assisted personal interview.

### Sample

Respondents living in England who were either of screening-eligible age (i.e. 60-70 years) or approaching screening-eligible age (i.e. 58-59 years) and had no CRC history were included in the ABACUS (*n* = 1568). Those aged 58-59 years (*n* = 187) were excluded from the present analysis as they were not yet eligible for CRC screening, leaving 1381 eligible respondents.

### Measures

#### FOBt screening uptake

FOBt screening uptake was assessed by self-report in the study interview with the question, ‘Have you ever been invited to do a stool test for the NHS Bowel Cancer Screening Programme’. Those who responded ‘Yes’ were asked how many kits they had received and how many they had completed. For this analysis, uptake was categorised dichotomously as ‘never’ vs ‘ever’ screeners.

#### Reading of the screening information

Respondents were shown an image of the front cover and first page of the standard NHS information booklet, *‘Bowel Cancer Screening: The Facts’*. They were asked how much of it they read when they received their screening invitation, with response options of ‘none’, ‘a little’, ‘some of it’, ‘most of it’, ‘almost all of it’, and ‘all of it’. Respondents who answered that they didn’t know or couldn’t remember how much they read were coded as having read ‘none’ (n = 20). Responses were dichotomised to reflect reading ‘none’ vs. ‘any’ of the booklet to identify individuals who failed to recall engaging with it, compared to those who recalled engaging with at least a little bit of it.

#### Sociodemographic factors

The sociodemographic factors assessed in the study interview were age (60-65 years; 66-70 years), sex (male; female), ethnicity (white; non-white) educational attainment (no formal qualifications; school level; degree level or higher), and occupation-based social grade according to the British National Readership Survey classification (A: high managerial, administrative, or professional; B: intermediate managerial, administrative, or professional; C1: supervisory, clerical and junior managerial, administrative, or professional; C2: skilled manual workers; D: semi and unskilled workers; E: state pensioners, casual or lowest grade workers, unemployed with state benefits).

#### Statistical analysis

Classic univariate statistics were used to describe characteristics of the study sample. The frequencies of sociodemographic characteristics and screening uptake status were tabulated against reading of *‘The Facts’* booklet (‘none’ vs. ‘any’). Multivariable-adjusted logistic regression was used to estimate odds ratios (ORs) and associated 95 % confidence intervals (CIs) for reading ‘*The Facts*’ (‘none’ vs. ‘any’) in ‘never’ vs. ‘ever’ screeners, adjusting for age, sex, ethnicity, education, and social grade. A sensitivity analysis was conducted, excluding the 20 participants who reported that they didn’t know or couldn’t remember how much of *‘The Facts’* booklet they had read. All statistical analyses were conducted using StataSE 13.1 (StataCorp, College Station, TX).

## Results

Of the 1381 eligible respondents, 74 (5 %) were missing data on one or more variable and were excluded, leaving 1307 respondents in the analytical sample. Table 1 shows the sample characteristics. Half of respondents (52 %; 681/1307) were aged 60-65 years and half (51 %; 664/1307) were male. Only 4 % (58/1307) were non-white. One-third of the sample had no formal educational qualifications (32 %; 416/1307), while one-fifth (19 %; 252/1307) had degree level education. One-quarter (25 %; 332/1307) were in social grades A/B (highest) and 23 % (295/1307) were in social grade E (lowest). Reported ‘ever’ uptake of FOBt screening in the national screening programme was 69 % (908/1307). Approximately one in five respondents reported that they had read none of *‘The Facts’* booklet (22 %; 287/1307); while just over half reported that they had read all of it (52 %; 680/1307; Table 1).

**Table 1:** Characteristics of the sample, England, 2014, n = 1307

Characteristic	N (%)
Age	
60-65	681 (52 %)
66-70	626 (48 %)
Sex	
Male	664 (51 %)
Female	643 (49 %)
Ethnicity	
White	1249 (96 %)
Non-white	58 (4 %)
Educational attainment	
Degree level or higher	252 (19 %)
School level	581 (44 %)
No formal qualifications	416 (32 %)
Missing	58 (4 %)
Social grade	
A/B (upper/middle class)	332 (25 %)
C1 (lower middle class)	289 (22 %)
C2 (skilled working class)	240 (18 %)
D (working class)	151 (12 %)
E (non-working/unemployed)	295 (23 %)
Amount read of ‘*The Facts*’	
None	287 (22 %)
A little	42 (3 %)
Some of it	85 (7 %)
Most of it	116 (9 %)
Almost all of it	97 (7 %)
Almost all of it	680 (52 %)
Screening uptake status	
‘Ever’	908 (69 %)
‘Never’	399 (31 %)

Table 2 shows the associations between FOBt screening uptake, sociodemographic factors and having read the *‘The Facts*’. Independently of sociodemographic factors, ‘never’ screeners were substantially more likely than ‘ever’ screeners to report having read none the booklet (63 % vs. 4 %; OR = 39.0; 95 % CI: 26.2-58.1; Table 2). Independently of screening uptake status and other sociodemographic factors, ‘non-white’ adults were more likely than ‘white’ adults to have read none of it (55 % vs. 20 %; OR = 3.47; 95 % CI: 1.60-7.54), as were those with no educational qualifications compared to those with a degree (26 % vs. 19 %) and those who were in the lowest social grade compared with the highest social grade (30 % vs. 17 %); although the adjusted ORs for these latter two variables were not statistically significant (Table 2).

**Table 2:** Associations between FOBt screening uptake, sociodemographic characteristics, and having read the standard information booklet about FOBt screening, England, 2014, *n* = 1307

	Read none of '*The Facts'* booklet	Adjusted OR* (95 % CI)
FOBt screening uptake status		
‘Ever’	36 (4 %)	1.00 (ref)
‘Never’	251 (63 %)	39.0 (26.2, 58.1)
Age		
60-65	171 (25 %)	1.00 (ref)
66-70	116 (19 %)	0.81 (0.56, 1.16)
Sex		
Female	154 (23 %)	1.00 (ref)
Male	133 (21 %)	1.34 (0.93, 1.92)
Ethnicity		
White	255 (20 %)	1.00 (ref)
Non-white	32 (55 %)	3.47 (1.60, 7.54)
Educational attainment		
Degree level or higher	49 (19 %)	1.00 (ref)
School level	122 (21 %)	1.14 (0.67, 1.95)
No formal qualifications	108 (26 %)	1.50 (0.82, 2.75)
Missing	8 (14 %)	0.77 (0.26, 2.26)
Social grade		
A/B (upper/middle class)	56 (17 %)	1.00 (ref)
C1 (lower middle class)	48 (17 %)	0.83 (0.47, 1.47)
C2 (skilled working class)	50 (21 %)	1.00 (0.54, 1.85)
D (working class)	44 (29 %)	1.35 (0.68, 2.67)
E (non-working/unemployed)	89 (30 %)	1.26 (0.72, 2.23)

*All variables in the table are mutually adjusted for in the logistic regression model. The ORs are for reading 'none' versus 'a little'/'some'/'most'/'almost all'/'all' of *The Facts* booklet

A sensitivity analysis was conducted, removing the 20 respondents who reported that they didn’t know or couldn’t remember how much of *‘The Facts’* booklet they had read. Of the remaining respondents, 267/1287 (21 %) had read none of the booklet, while 1020/1287 (79 %) had read at least a little bit of it. The multivariable-adjusted OR for reading none of *‘The Facts’* became much stronger in magnitude in this analysis (OR = 60.97; 95 % CI: 37.87-98.18). This change might be because the majority of those who didn’t know or couldn’t remember how much they had read were ‘ever’ screeners (14/20; 70 %). The multivariable-adjusted ORs for the associations between sociodemographic factors and the amount of the booklet read were negligibly changed (not shown).

## Discussion

In this population-based survey of FOBt screening-eligible English adults, nearly two in three screening non-attenders reported that they had read none of standard informational material, compared with less than one in twenty screening attenders. This finding is concerning, given that ‘*The Facts*’ is meant to inform the public about the risks and benefits of screening and to support informed decision-making in advance of receiving the FOBt kit for screening. A substantial proportion of the screening-eligible population may not have benefited from the information made available in ‘*The Facts*’ booklet. We observed ethnic, educational, and social grade-based inequalities in self-reported reading of the booklet, which mirrored previously observed social inequalities in actual uptake of the screening test [11, 23, 24]. This finding raises the question of whether unequal engagement with available information might contribute to inequalities in screening participation. Future research should investigate this possibility.

Previous research has identified that low health literacy is associated with non-participation in CRC screening in England and elsewhere [6–11]. Adults with low health literacy have been shown to experience a greater burden of information processing when reading ‘*The Facts*’ [8]. Low numeracy or numerical skill, which is correlated with low health literacy, has also been identified as a direct barrier to reading ‘*The Facts*’ [25]. These findings indicate that ‘never’ screeners may disproportionately experience literacy or numeracy barriers, which may discourage them from reading ‘*The Facts*’. A limitation is that we do not know what proportion of ‘never’ screeners may have had made an informed choice not to take part in screening prior to receiving the invitation. Whether reading *The Facts* is necessary to make an informed decision about FOBt screening is unknown, although it represents a key public communication opportunity for the NHS that should not be differentially accessible across the screening-eligible population. In future, the reasons why some people do not read ‘*The Facts*’ booklet must be better understood in order to improve public engagement with information materials.

This study has limitations. Uptake of FOBt screening was self-reported, which may introduce recall errors and subsequent bias into the results of the study. The self-reported rates of ‘ever’ FOBt screening were validated against records from the NHS Bowel Cancer Screening Programme in a subset of 516 respondents and found to be highly concordant (94.2 % agreement, k = 0.74) [22]. The proportion of self-reported ‘ever’ FOBt uptake in this study (69 %) was consistent with the NHS record of 70 % for ‘ever’ screening in England during a similar time frame [3]. However, if any randomly distributed recall errors were present, they would have biased the ORs towards the null. If participants who had read *‘The Facts’* booklet were more likely to accurately recall whether they had completed screening than those who had not read the booklet, the ORs may be biased in a direction dependent on the degree of error in responses among those who did not read the booklet. Reading of *‘The Facts’* was also assessed by self-report; accuracy of its recall could have been differential according to screening uptake status. It is difficult to assess the degree to which this may have occurred; but, if those who took up screening were more likely to recall reading the booklet than those who did not, the ORs could be biased upwards.

We were statistically underpowered to detect some associations with sociodemographic factors, but we did observe important social trends in engagement with ‘*The Facts*’ booklet that deserve further investigation. Although response rates could not be monitored due to the sampling methods, the sample was population-representative of this age group in England according to comparison against figures from the 2011 Census [26]. This article presents a secondary analysis of the ABACUS dataset, and therefore we did not have more in-depth measures about what parts of the booklet that respondents may or may not have read, and we did not ask the reasons why some respondents did not read the booklet. Future research should examine why some people do or do not engage with the screening information materials and the degree to which standardised information materials influence people’s decisions to participate or not in screening.

## Conclusions

Resolving the issue of low information engagement among non-attenders will be more complicated than the simple provision of plain language, as is currently done in the FOBt booklet. There is an established and legitimate emphasis in the NHS on ensuring that people participating in cancer screening have made an informed choice to do so, although more needs to be done to ensure the same for those who abstain from screening. It is time for screening delivery to be better tailored to the communities being served. Individuals with low literacy or numeracy may never be effectively reached through mailed written materials alone, even when presented in simplified ‘plain English’ or in plain language translations [8, 16, 27]. Automated voice and text-message reminders, patient navigation, and shared decision making methods may be effective to engage people with CRC screening [28–34]. The degree to which public engagement with the decision-making process about cancer screening is socially unequal must be better understood so that comprehensive and equitable public communication strategies can be developed.

## Abbreviations

ABACUS, Attitudes, Behaviour, and Cancer UK Survey; CRC, colorectal cancer; FOBt, faecal occult blood test; NHS, National health service; UK, United Kingdom
